# Supplementary material for: Cu-based high-entropy two-dimensional oxide as stable and active photothermal catalyst
Source: Nat Commun. 2023 Jun 1;14:3171. doi: 10.1038/s41467-023-38889-5 (PMC10235064; doi:10.1038/s41467-023-38889-5)
Supplement: Supplementary file 1 — Supplementary Information [file 41467_2023_38889_MOESM1_ESM.pdf]

Cu-based high-entropy two-dimensional oxide as stable and active photothermal catalyst

Yaguang Li[1,2,*,✉], Xianhua Bai[1,*], Dachao Yuan[2,*], Chenyang Yu[1], Xingyuan San[1], Yunna Guo[3], Liqiang Zhang[3,✉], Jinhua Ye[1,4,5,✉]

[1]Research Center for Solar Driven Carbon Neutrality, Hebei Key Lab of Optic-electronic Information and Materials, The College of Physics Science and Technology, Institute of Life Science and Green Development, Hebei University, Baoding, 071002, China.

[2]College of Mechanical and Electrical Engineering, Key Laboratory Intelligent Equipment and New Energy Utilization of Livestock and Poultry Breeding, Hebei Agricultural University, Baoding 071001, China.

[3]Clean Nano Energy Center, State Key Laboratory of Metastable Materials Science and Technology, Yanshan University, Qinhuangdao 066004, China.

[4]International Center for Materials Nanoarchitectonics (WPI-MANA), National Institute for Materials Science (NIMS), 1-1 Namiki, Tsukuba, Ibaraki 305-0044, Japan.

[5]Graduate School of Chemical Science and Engineering, Hokkaido University, Sapporo 060-0814, Japan.

*These authors contributed equally to this work.

✉Correspondence and requests for materials should be addressed to Y. Li (email: liyaguang@hbu.edu.cn), L. Zhang (email: lqzhang@ysu.edu.cn) or J. Ye. (email: Jinhua.YE@nims.go.jp).

Supplementary Methods

The homemade TiC/Cu-based device

Magnetron sputtering was used to deposit TiC/Cu film on the reaction tube. TiC and Cu were used as targets and the working gas was Ar with 99.99% purity. Before the deposition process, the reaction tube was washed with deionized water, acetone and ethanol subsequently, and then the glow-discharge was used to clean the reaction tube. The Cu layer and TiC film were orderly deposited on the surface of the tube by magnetron sputtering of SP-0707AS. Specific parameters: the power was 4.5 kW, the sputtering pressure was 6×10^{-2} Pa, the bias voltage was 150 V, the sputtering temperature was 200 °C, and the sputtering time for Cu layer, TiC film was 16 min, 4 min, respectively. The follow glass vacuum layer was provided by Hebei scientist research experimental and equipment trade Co., Ltd. with 1×10^{-3} Pa of pressure.

Characterizations

The overall composition of the prepared samples was studied by the powder X-ray diffraction (XRD), which was performed on a Bede D1 system operated at 20 kV and 30 mA with Cu Kα radiation (λ= 1.5406 Å). The scanning electron microscopy (SEM) images were tested with the FEI Nova NanoSEM450 (Czech Republic). Transmission electron microscopy (TEM, ARM 200 F and JEOL F200+) was used to identify the morphology and the crystal structure of the nanostructures. Thermo ESCALAB-250 spectrometer with a monochromatic Al Kα radiation source (1486.6 eV) was used to detect the valence state of materials. Zennium_Pro (Zahner, Germany) was an electrochemical workstation. N_2-sorption isotherms were collected on a Belsorb-Max system. Brunauer-Emmett-Teller (BET) specific surface areas were calculated from adsorption data. The thickness of catalysts was tested by AFM (MFP-3D Origin+, Oxford Instruments).

Computational method

We have used the Vienna Ab Initio Package (VASP) to perform all the density functional theory (DFT) calculations within the generalized gradient approximation (GGA) using the PBE formulation.[1,2] We have chosen the projected augmented wave (PAW) potentials[3] to describe the ionic cores and accounted for valence electrons into account using a plane wave basis set with a kinetic energy cutoff of 450 eV. Partial occupancies of the Kohn−Sham orbitals were allowed using the Gaussian smearing method and a width of 0.05 eV. The on-site corrections (DFT+U) were applied to the 4f electron of Ce atoms (U_{eff}=5.0 eV) in CeO_2 and 3d electron of Cu atom (U_{eff}=7.0 eV) by the approach from Dudarev et al.[4] The electronic energy was considered self-consistent when the energy change was smaller than 10^{-5} eV. A geometry optimization was considered convergent when the force change was smaller than 0.02 eV/Å. Grimme's DFT-D3 methodology was used to describe the dispersion interactions.[5]

The equilibrium lattice constant of CeO_2 unit cell was optimized by using an 11×11×11 Monkhorst-Pack k-point grid for Brillouin zone sampling, to be a=5.479 Å. We then use it to construct a CeO_2(111) surface model (model 0) with *p*(4×3) periodicity in the X and Y directions and three stoichiometric layers in the Z direction by vacuum depth of 15 Å in order to separate the surface slab from its periodic duplicates. This model comprises of 36 Ce and 72 O atoms. Model 1 was built by doping 8 Cu atoms and 4 Zn atoms into model 0 and removing 12 O atoms in order to balance electrons. Model 2 was built by doping 8 Cu, 4 Zn, 2 Al and 2 Zr atoms into model 0 and removing 12 O atoms in order to balance electrons. During structural optimizations, a 1×2×1 Brillouin zone was used for k-point sampling, and the bottom stoichiometric layer was fixed while the rest were allowed to fully relax.

The equilibrium lattice constant of cubic CuO unit cell was optimized when using an 11×11×11 Monkhorst-Pack k-point grid for Brillouin zone sampling, to be a=4.152 Å. We then use it to construct a CuO(111) surface model (model 3) with *p*(2×2) periodicity in the X and Y directions and four stoichiometric layers in the Z direction by vacuum depth of 15 Å in order to separate

the surface slab from its periodic duplicates. Model 3 comprises of 64 Cu and 64 O atoms. During structural optimizations, a 2×2×1 in the Brillouin zone was used for k-point sampling, and the bottom two stoichiometric layers were fixed while the rest were allowed to fully relax.

The adsorption energy (E_{ads}) of adsorbate A was defined as

$$E_{ads} = E_{A/surf} - E_{surf} - E_{A(g)}$$

Where $E_{A/surf}$, E_{surf} and $E_{A(g)}$ are the energy of adsorbate A adsorbed on the polyimide, the energy of clean polyimide, and the energy of isolated A molecule in a cubic periodic box with a side length of 20 Å and a 1×1×1 Monkhorst-Pack k-point grid for Brillouin zone sampling, respectively.

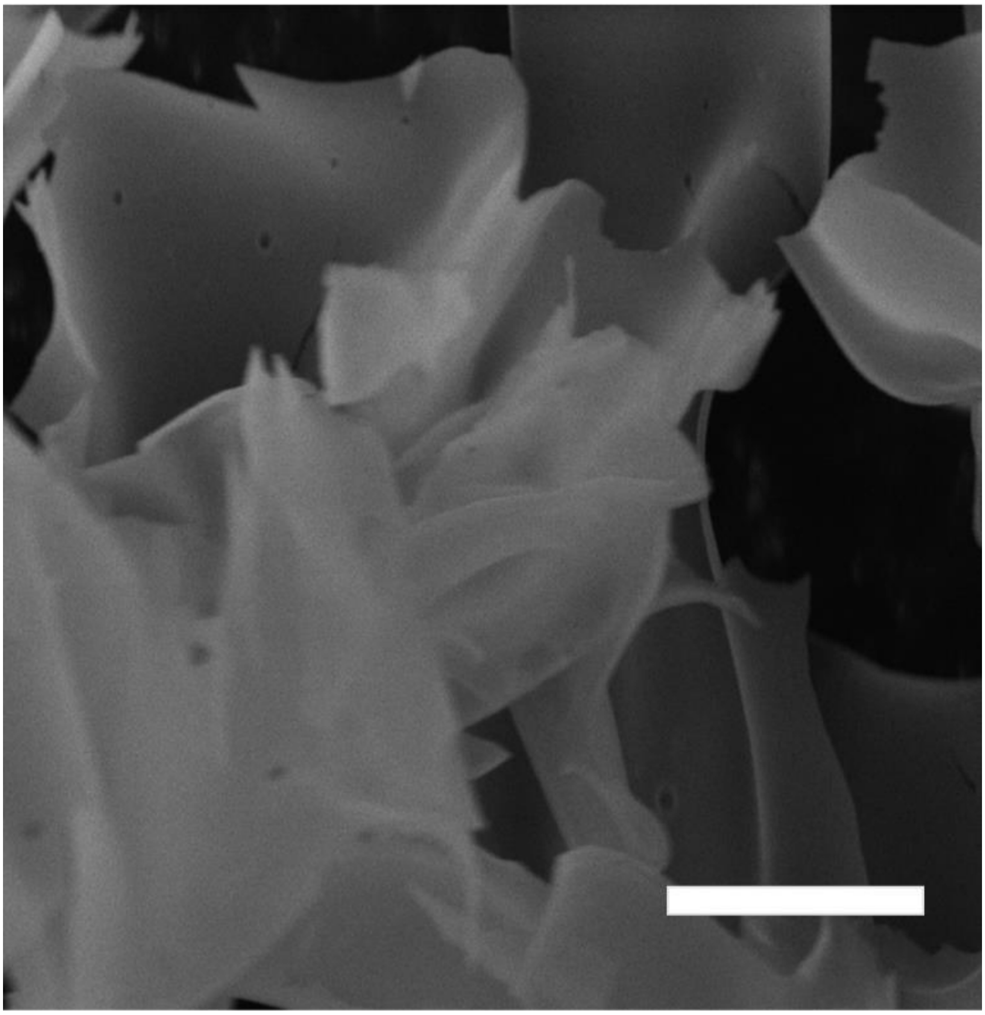

Supplementary Fig. 1 | SEM image of PVP micelle after the freeze-drying process. The scale bar is 2μm.

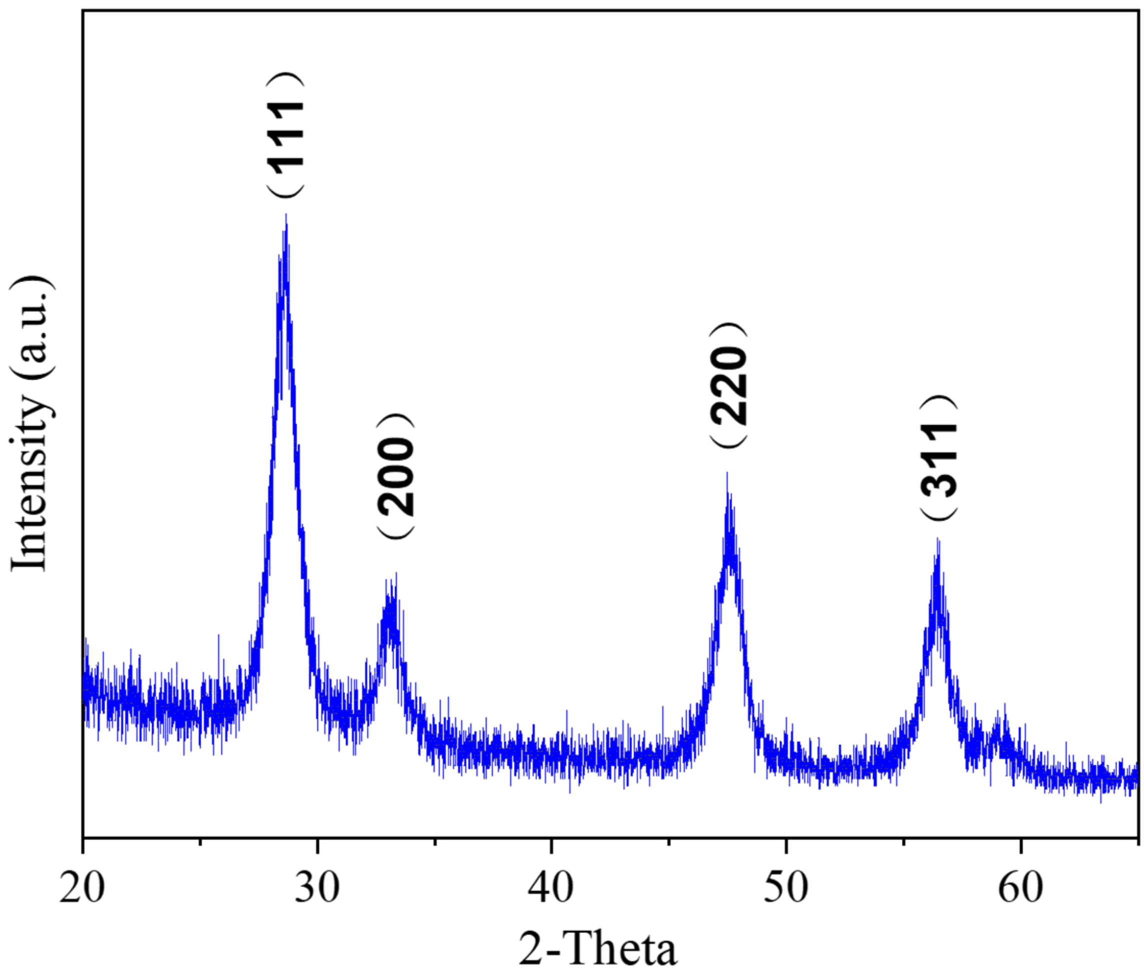

Supplementary Fig. 2 | XRD pattern of 2D $Ce_1Cu_1Mn_1Mg_1Al_1Co_1La_1Zr_1Ca_1Y_1O_x$.

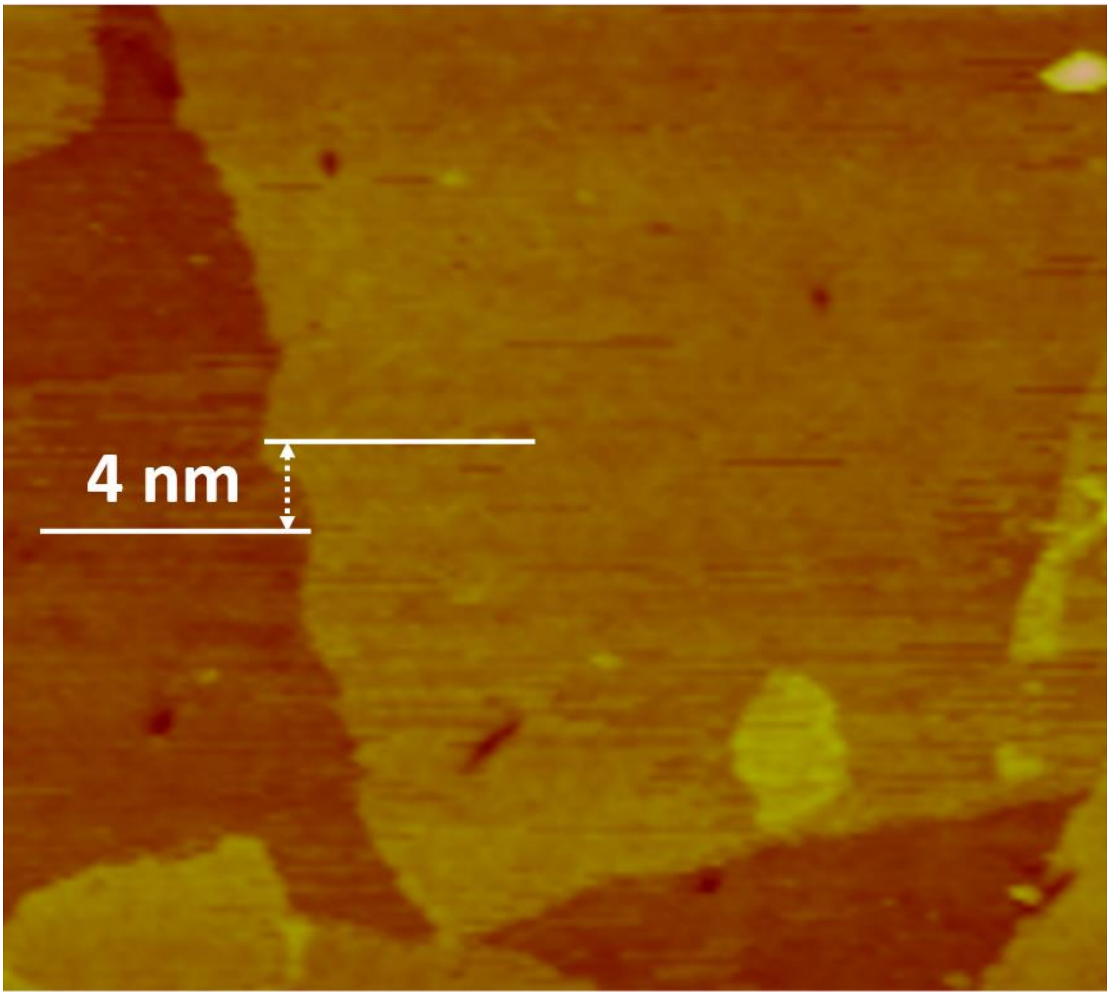

Supplementary Fig. 3 | The AFM image of 2D $Cu_2Zn_1Al_{0.5}Ce_5Zr_{0.5}O_x$.

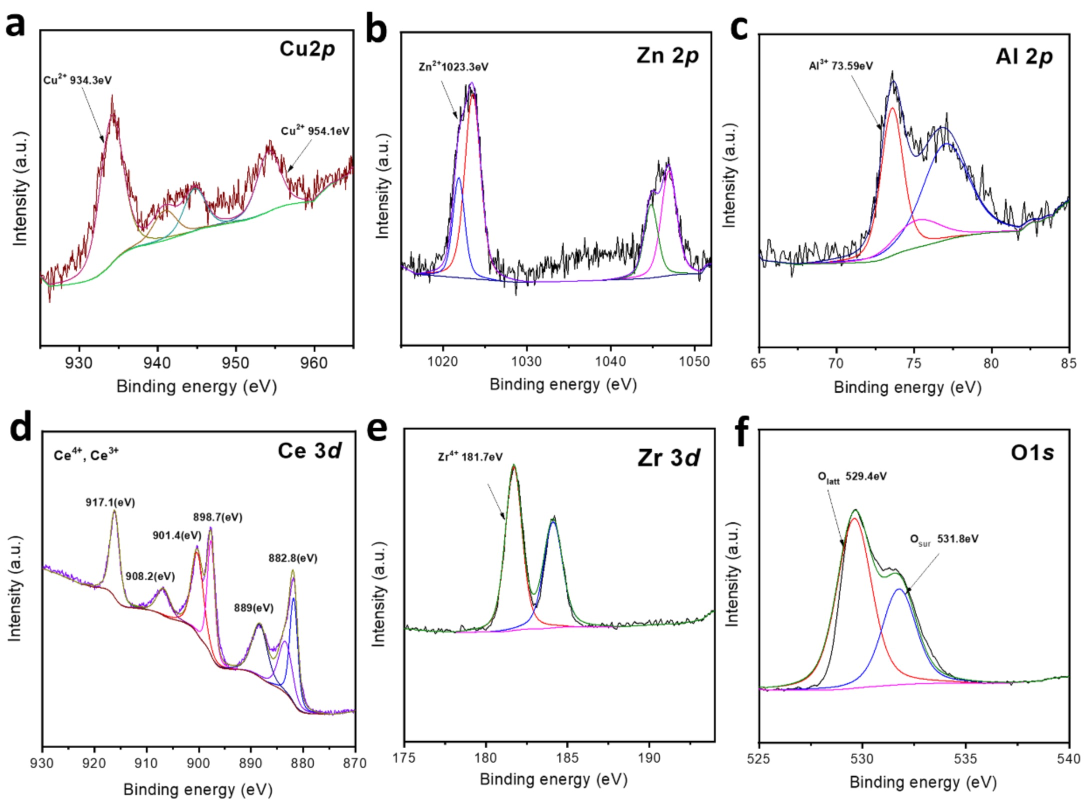

Supplementary Fig. 4 | The XPS analysis of 2D $Cu_2Zn_1Al_{0.5}Ce_5Zr_{0.5}O_x$. **a-f** The Cu2*p*, Zn 2*p*, Al 2*p*, Ce 3*d*, Zr 3*d*, O 1*s* XPS spectra of 2D $Cu_2Zn_1Al_{0.5}Ce_5Zr_{0.5}O_x$.

The XPS analysis were referred by the reported literatures of Cu,[6] Zn,[7] Al,[8] Ce,[9] Zr,[10] O.

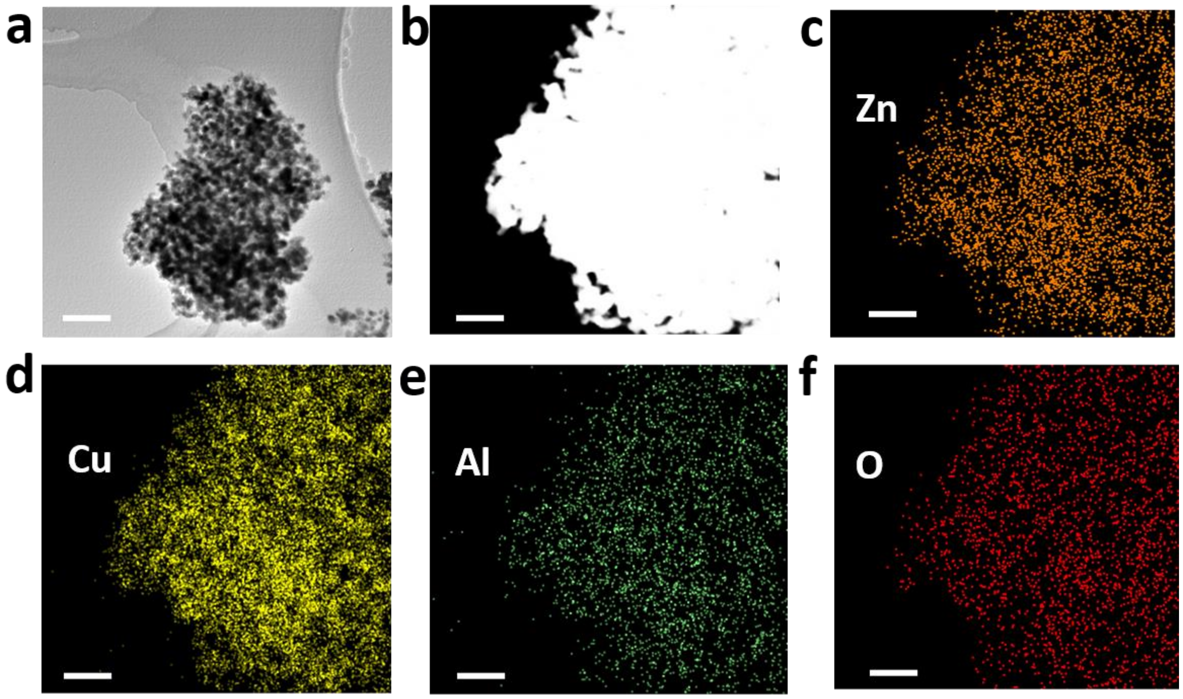

Supplementary Fig. 5 | The characterizations of $Cu_6Zn_3Al_1$. **a** TEM image, **b** STEM image **c-f** Zn, Cu, Al, O elemental mapping images of $Cu_6Zn_3Al_1$. The scale bars in **a, b-f** are 200 nm, 100 nm, respectively.

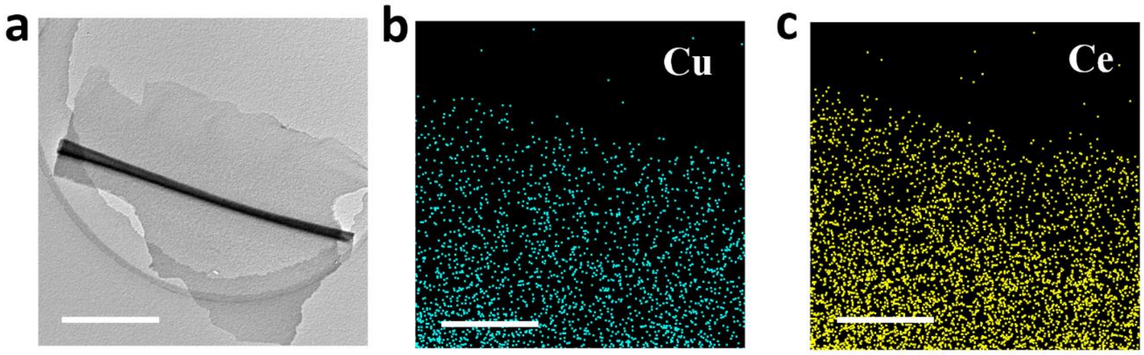

Supplementary Fig. 6 | The characterizations of 2D $Cu_2Ce_7O_x$. **a** TEM image, **b-c** Cu, Ce elemental mapping images of 2D $Cu_2Ce_7O_x$. The scale bars in **a, b-c** are 500 nm, 250 nm, respectively.

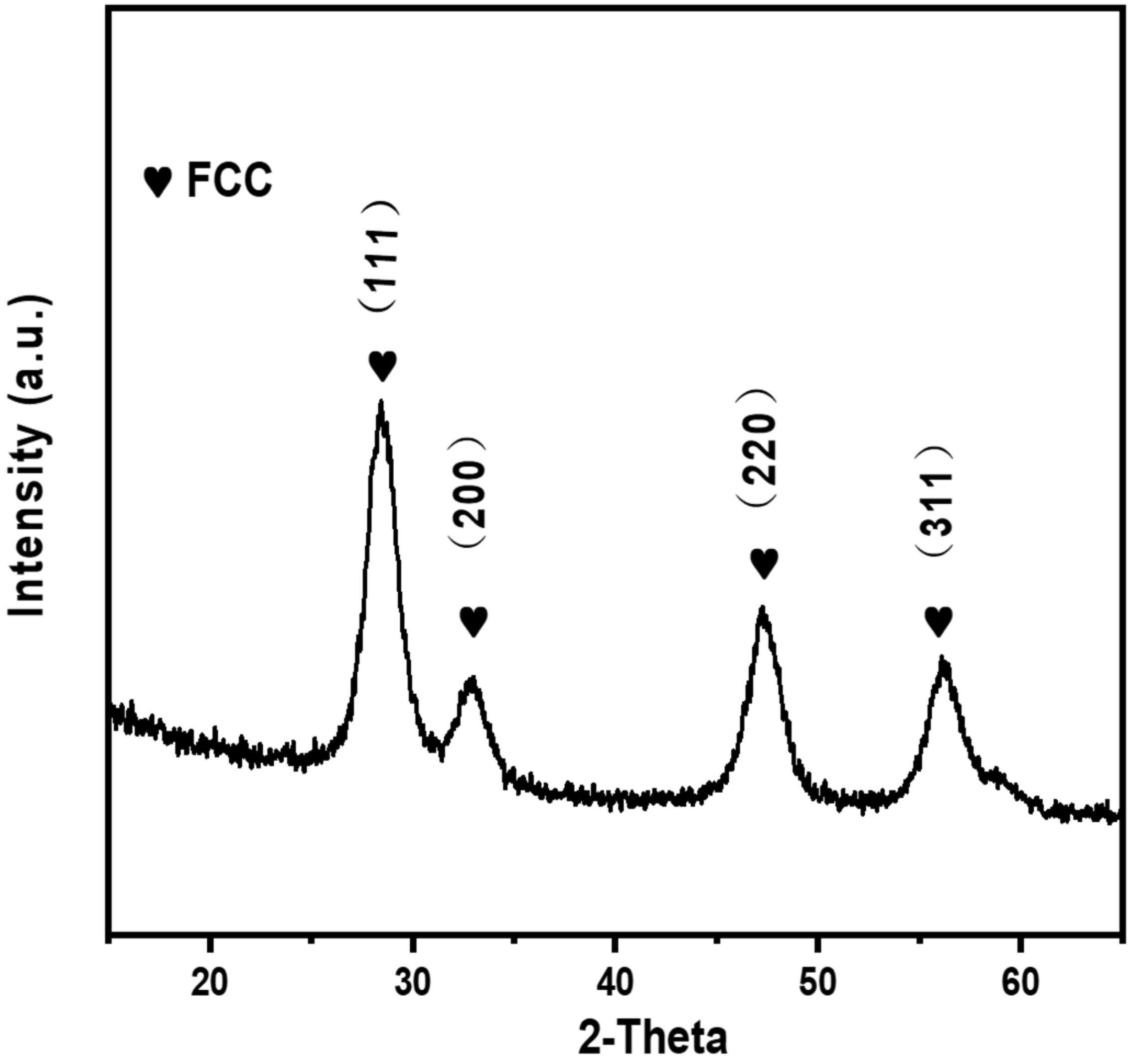

Supplementary Fig. 7 | XRD pattern of 2D $Cu_2Ce_7O_x$.

The XRD pattern of 2D $Cu_2Ce_7O_x$ was similar to that of CeO_2.[11,12]

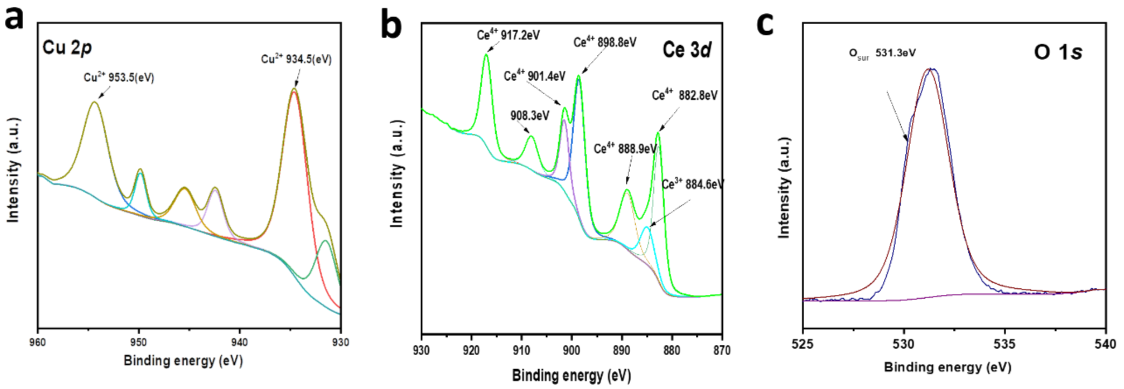

Supplementary Fig. 8 | The XPS characterization of 2D $Cu_2Ce_7O_x$. **a-c** The Cu2*p*, Ce 3*d*, O 1*s* XPS spectra of 2D $Cu_2Ce_7O_x$.

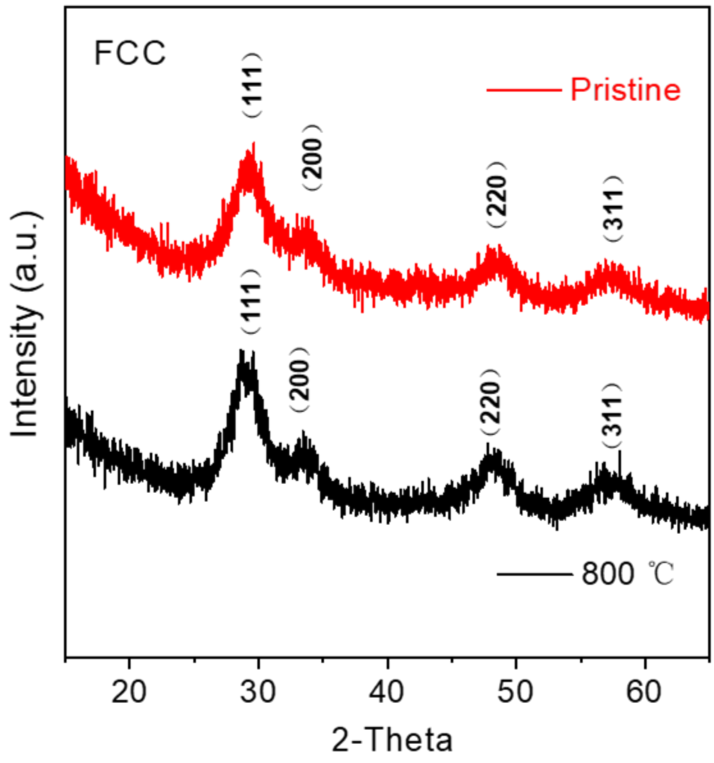

Supplementary Fig. 9 | The XRD patterns of pristine 2D $Cu_2Zn_1Al_{0.5}Ce_5Zr_{0.5}O_x$ and 2D $Cu_2Zn_1Al_{0.5}Ce_5Zr_{0.5}O_x$ after 800 °C of RWGS.

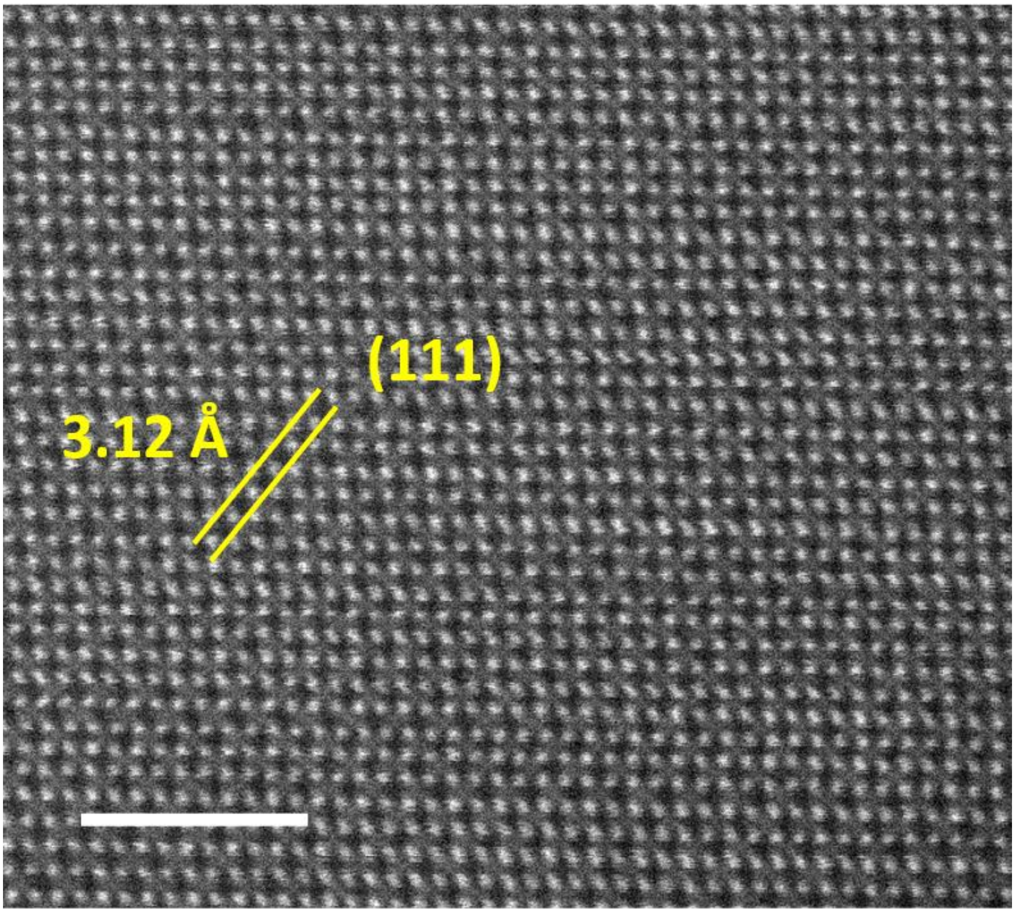

Supplementary Fig. 10 | The HAADF-STEM image of 2D $Cu_2Zn_1Al_{0.5}Ce_5Zr_{0.5}O_x$ after 800 °C of in-situ experiment. The scale bar is 2 nm.

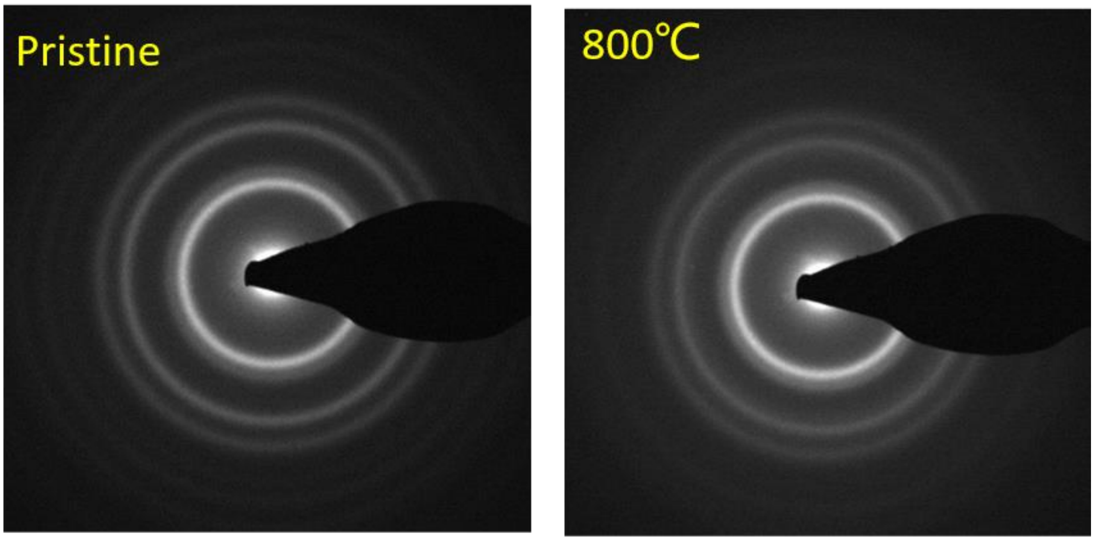

Supplementary Fig. 11 | Electron diffraction patterns of pristine 2D $Cu_2Zn_1Al_{0.5}Ce_5Zr_{0.5}O_x$ and 2D $Cu_2Zn_1Al_{0.5}Ce_5Zr_{0.5}O_x$ after 800 °C of in-situ RWGS experiment.

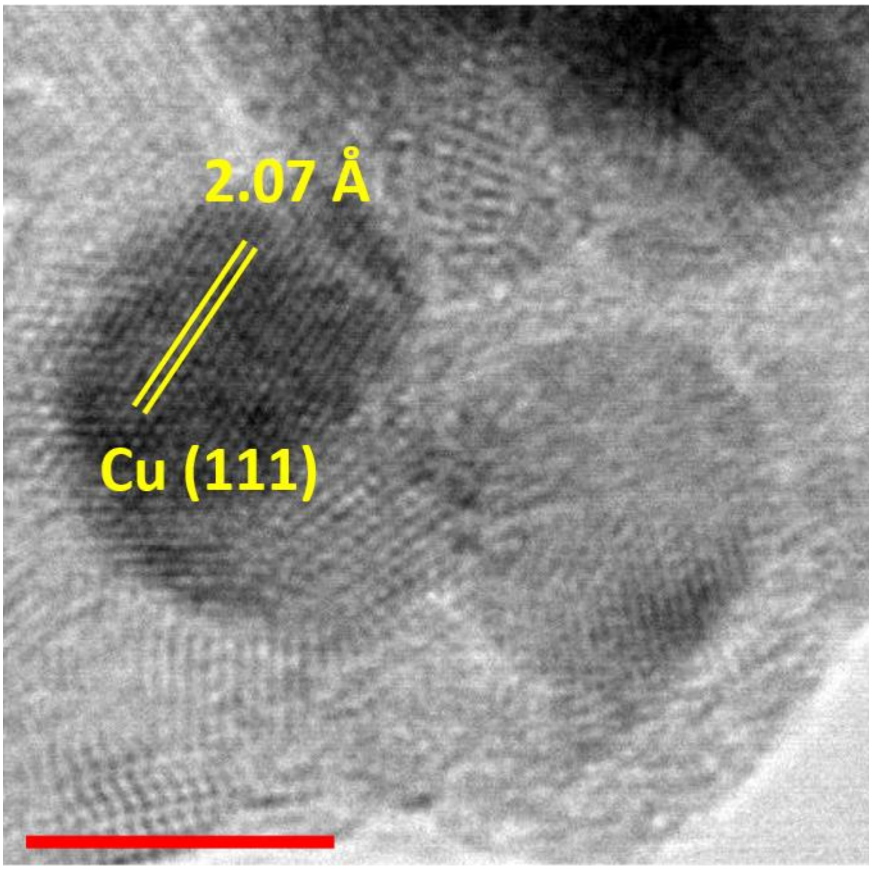

Supplementary Fig. 12 | The HRTEM image of 2D $Cu_2Ce_7O_x$ at 400 °C of in-situ experiment.

The scale bar is 5 nm.

The 2.07 Å of lattice spacing was corresponding to the (111) plane of metallic Cu.[13]

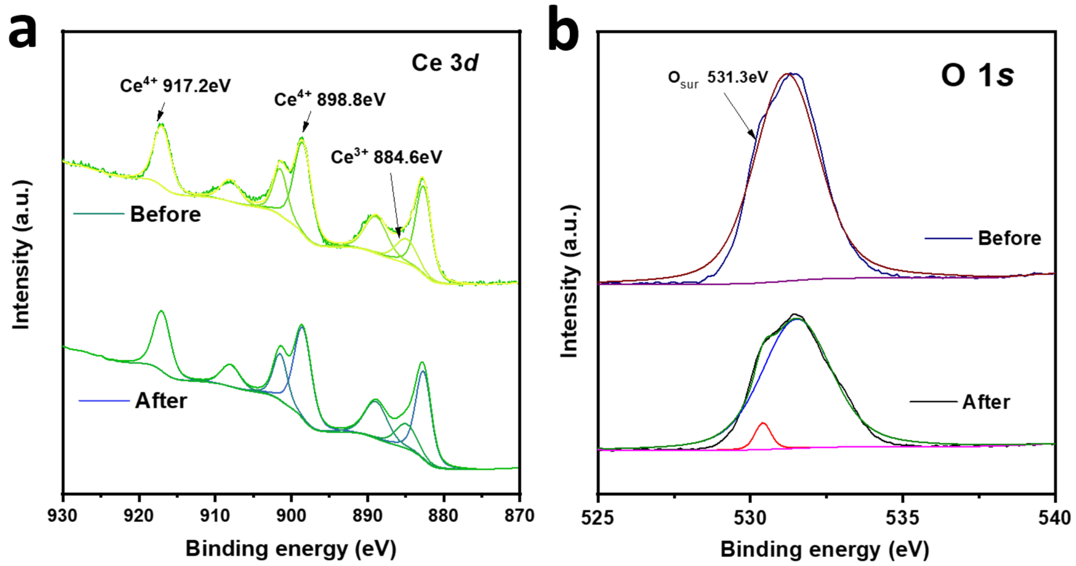

Supplementary Fig. 13 | The XPS characterizations of 2D $Cu_2Ce_7O_x$ before and after H_2-TPR.

a-b The Ce 3*d*, O 1*s* XPS spectra of 2D $Cu_2Ce_7O_x$ before and after H_2-TPR.

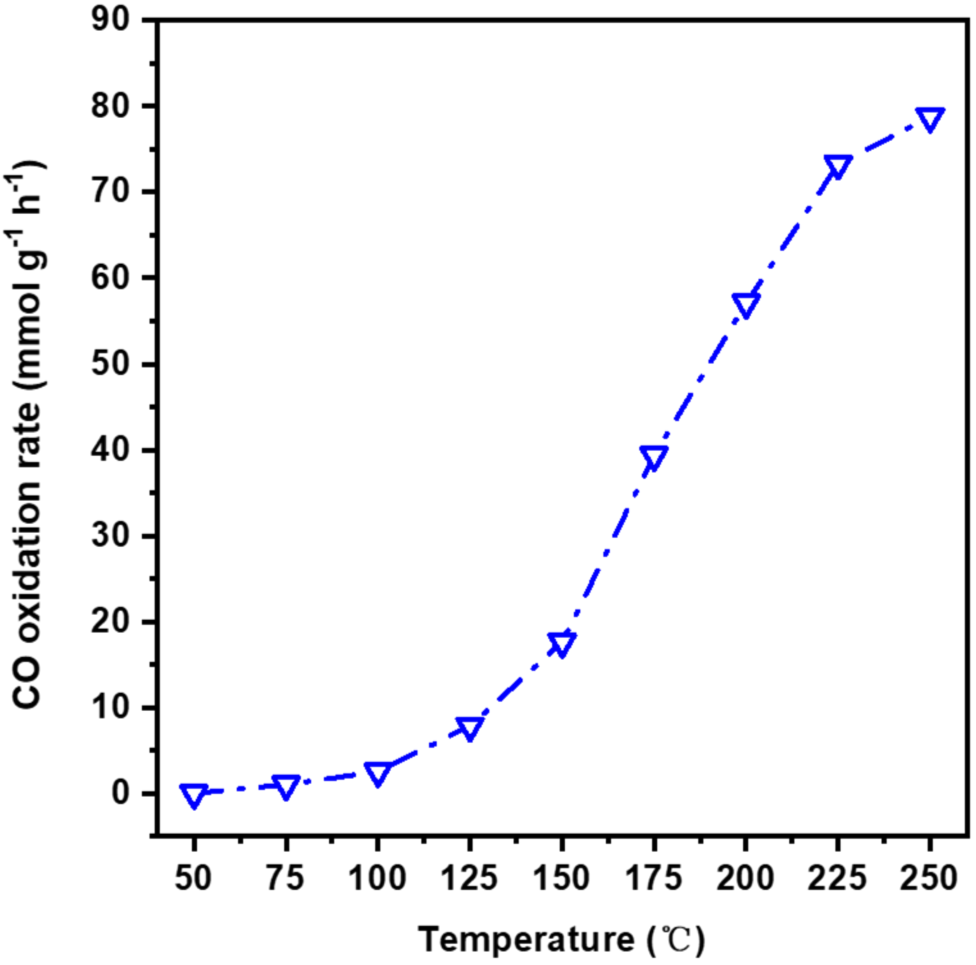

Supplementary Fig. 14 | The CO oxidation performance of 2D $Cu_2Zn_1Al_{0.5}Ce_5Zr_{0.5}O_x$.

The CO oxidation was tested by the fixed-bed reactor (XM190708-007, DALIAN ZHONGJIARUILIN LIQUID TECHNOLOGY CO., LTD) in continuous flow form. Typically, 15 mg of 2D $Cu_2Zn_1Al_{0.5}Ce_5Zr_{0.5}O_x$ was placed in a quartz flow reactor and the feeding gas of $CO/O_2/Ar/N_2$= 1/20/99/80 with 100 sccm of flow rate was regulated by the mass flow controller. The reaction products were tested by gas chromatography (GC) 7890A equipped with FID and TCD detectors.

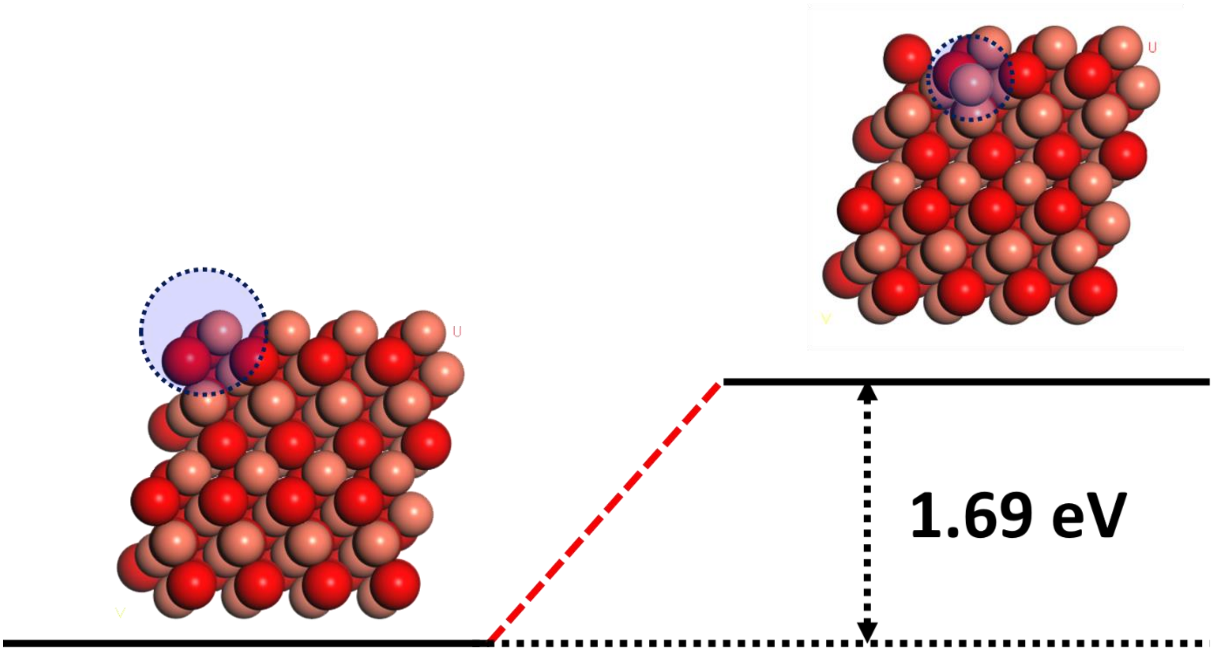

Supplementary Fig. 15 | Atomic structures of CuO before and after metallic Cu precipitation with corresponding free energy change.

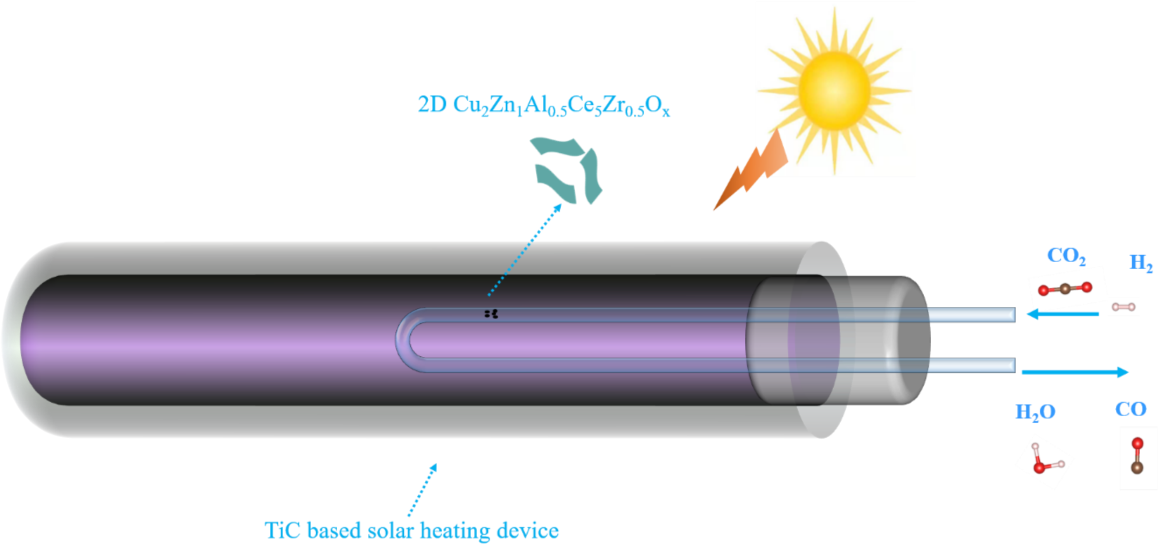

Supplementary Fig. 16 | Schematic of TiC-based solar heating device loaded with 2D $Cu_2Zn_1Al_{0.5}Ce_5Zr_{0.5}O_x$ for solar heating RWGS.

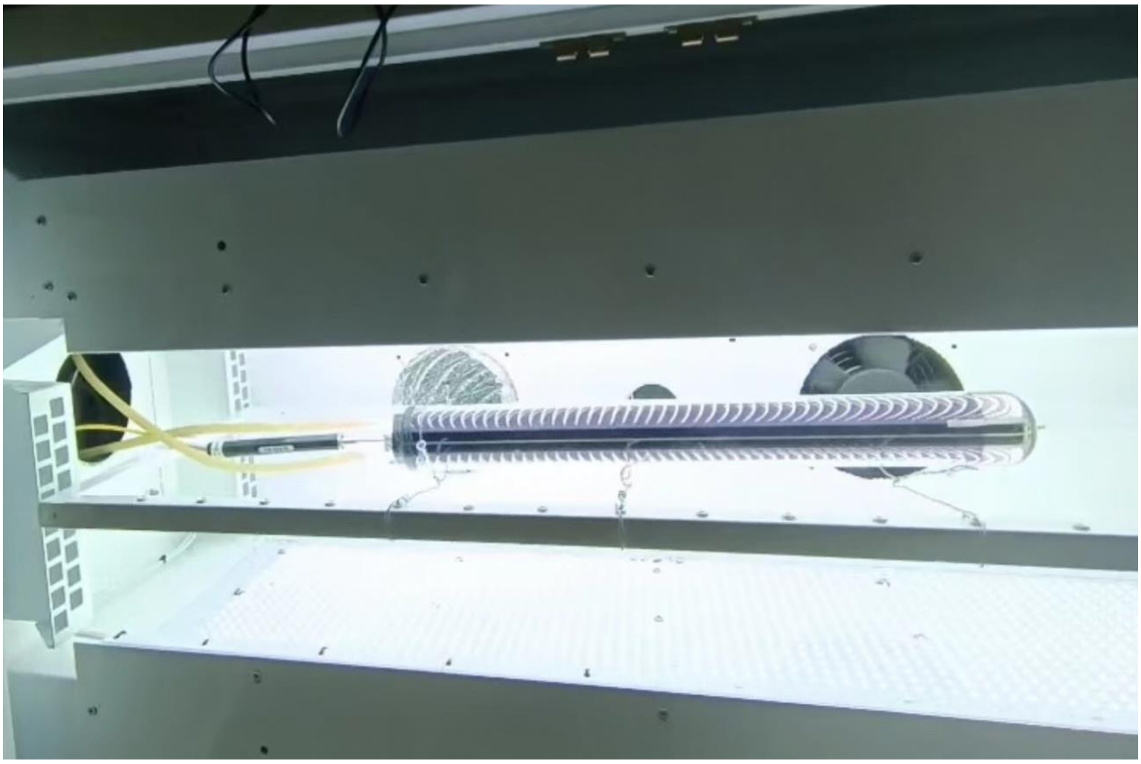

Supplementary Fig. 17 | Photograph of TiC-based solar heating device loaded with 100 g 2D $Cu_2Zn_1Al_{0.5}Ce_5Zr_{0.5}O_x$ under double side irradiation.

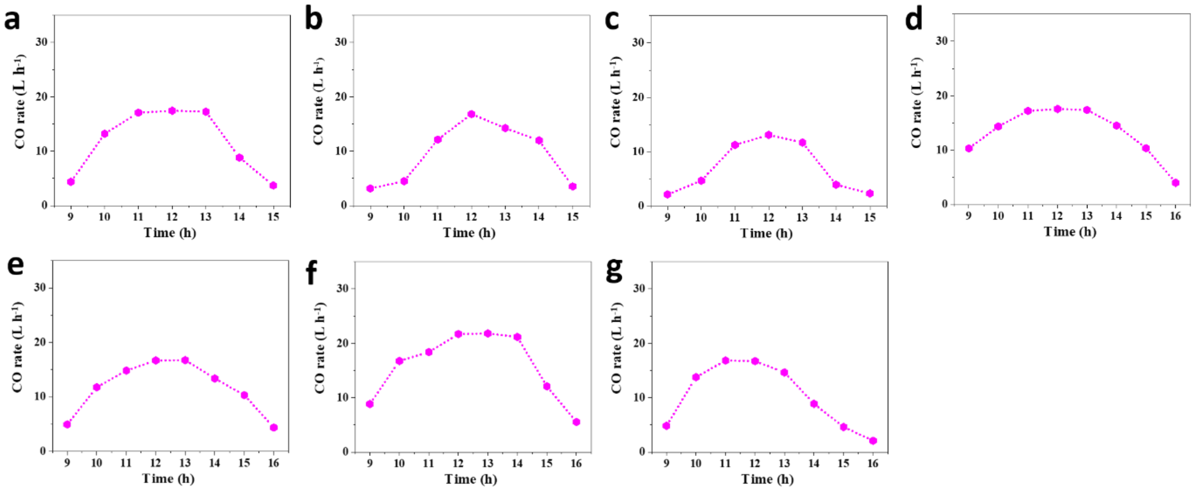

Supplementary Fig. 18 | The RWGS performance under outdoor sunlight irradiation. **a-g** The CO generation rate of solar heating RWGS through TiC-based solar heating device loaded with 100 g 2D $Cu_2Zn_1Al_{0.5}Ce_5Zr_{0.5}O_x$ under ambient sunlight irradiation, on December 12, 13, 14, 17, 18, 20, 21, 2021, in Baoding City, China.

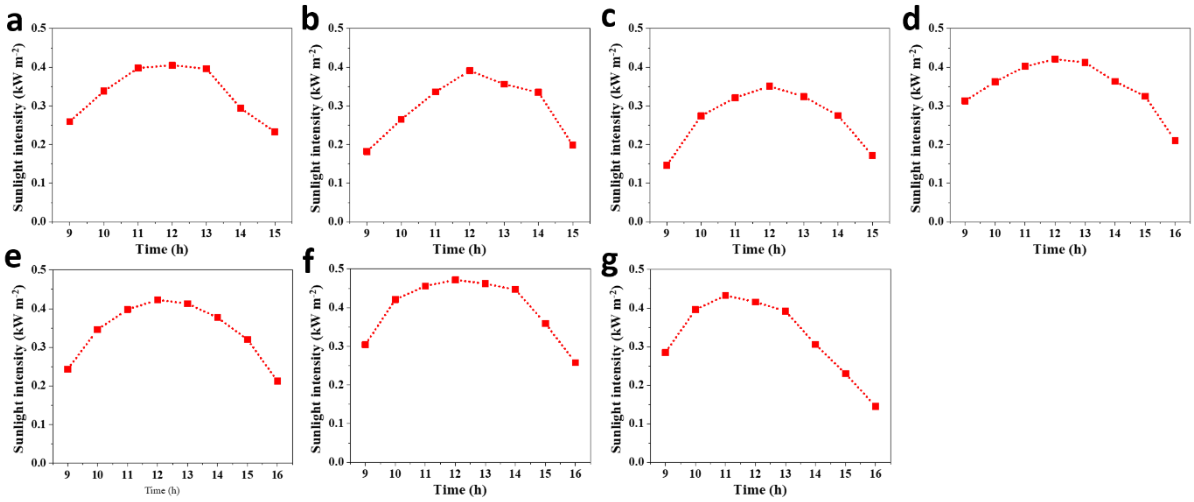

Supplementary Fig. 19 | The outdoor sunlight condition. **a-g** The outdoor sunlight intensity on December 12, 13, 14, 17, 18, 20, 21, 2021, in Baoding City, China.

Supplementary References

1 Kresse, G. & Furthmüller, J. Efficiency of ab-initio total energy calculations for metals and semiconductors using a plane-wave basis set. *Comp. Mater. Sci.* **6**, 15-50 (1996).

2 Kresse, G. & Furthmüller, J. Efficient iterative schemes for ab initio total-energy calculations using a plane-wave basis set. *Phy. Rev. B* **54**, 11169-11186 (1996).

3 Perdew, J. P., Burke, K. & Ernzerhof, M. Generalized Gradient Approximation Made Simple. *Phy. Rev. Lett.* **77**, 3865-3868 (1996).

4 Dudarev, S. L., Botton, G. A., Savrasov, S. Y., Humphreys, C. J. & Sutton, A. P. Electron-energy-loss spectra and the structural stability of nickel oxide: An LSDA+U study. *Phys. Rev. B* **57** (1998).

5 Grimme, S., Antony, J., Ehrlich, S. & Krieg, H. A consistent and accurate ab initio parametrization of density functional dispersion correction (DFT-D) for the 94 elements H-Pu. *J. Chem. Phy.* **132**, (2010).

6 Halder, A. *et al.* CO_2 Methanation on Cu-Cluster Decorated Zirconia Supports with Different Morphology: A Combined Experimental In Situ GIXANES/GISAXS, Ex Situ XPS and Theoretical DFT Study. *ACS Catal.* **11**, 6210-6224 (2021).

7 Correia, F. C. *et al.* XPS analysis of ZnO:Ga films deposited by magnetron sputtering: Substrate bias effect. *Appl. Surf. Sci.* **458**, 1043-1049 (2018).

8 Zhang, L. *et al.* Decontamination of U(VI) on graphene oxide/Al_2O_3 composites investigated by XRD, FT-IR and XPS techniques. *Environ. Pollut.* **248**, 332-338 (2019).

9 López Cámara, A. *et al.* Novel manganese-promoted inverse CeO_2/CuO catalyst: In situ characterization and activity for the water-gas shift reaction. *Catal. Today* **339**, 24-31 (2020).

10 Li, H. *et al.* CO_2 activation on ultrathin ZrO_2 film by H_2O co-adsorption: In situ NAP-XPS and IRAS studies. *Surf. Sci.* **679**, 139-146 (2019).

11 Sarkar, A. *et al.* Determining role of individual cations in high entropy oxides: Structure and reversible tuning of optical properties. *Scr. Mater.* **207**, 114273 (2022).

12 Spiridigliozzi, L., Ferone, C., Cioffi, R. & Dell'Agli, G. A simple and effective predictor to design novel fluorite-structured High Entropy Oxides (HEOs). *Acta Mater.* **202**, 181-189 (2021).

13 Chen, T.-A. *et al.* Wafer-scale single-crystal hexagonal boron nitride monolayers on Cu (111). *Nature* **579**, 219-223 (2020).
